# Appointment structure in Malaysian healthcare system during the COVID-19 pandemic: The public perspective

**DOI:** 10.1186/s12913-021-07456-3

**Published:** 2022-02-03

**Authors:** Ramani Subramaniam Kalianan, Yuan Liang Woon, Yee Liang Hing, Chin Tho Leong, Wei Yin Lim, Ching Ee Loo, Lee Lan Low

**Affiliations:** 1 Centre for Clinical Epidemiology, Institute for Clinical Research, National Institutes of Health, Ministry of Health, Block B4, Jalan Setia Murni, U13/52, Seksyen U13, Setia Alam, 40170 Shah Alam, Selangor Malaysia; 2 Centre for Health Services Research, Institute for Health Systems Research, National Institutes of Health, Ministry of Health, Block B2, Jalan Setia Murni, U13/52, Seksyen U13, Setia Alam, 40170 Shah Alam, Selangor Malaysia

**Keywords:** Public, Physical distancing, Clinic, Appointment, Malaysia, Healthcare, COVID-19

## Abstract

**Introduction:**

Evidence shows physical distancing of one metre or more is important to reduce person-to-person SARS-CoV-2 transmission. This puts the Malaysian public healthcare system to a test when overcrowding has always been an issue. A new clinical appointment structure was proposed in the Malaysian public healthcare system amidst the pandemic to reduce the transmission risk. We aim to explore the general public’s view on the proposed clinic appointment structure.

**Methods:**

A cross-sectional anonymous web-based survey was conducted between 10th September 2020 and 30th November 2020. The survey was open to Malaysian aged 18 years and older via various social media platforms. The questionnaire consists of sociodemographic, experience of utilising healthcare facilities, and views on clinic appointment structure.

**Results:**

A total of 1,144 complete responses were received. The mean age was 41.4 ± 12.4 years and more than half of the respondents had a preference for public healthcare. Among them, 77.1% reported to have a clinical appointment scheduled in the past. Less than a quarter experienced off-office hour appointments, mostly given by private healthcare. 70.2% answered they would arrive earlier if they were given a specific appointment slot at a public healthcare facility, as parking availability was the utmost concern. Majority hold positive views for after office hour clinical appointments, with 68.9% and 63.2% agreed for weekend and weekday evening appointment, respectively. The top reason of agreement was working commitment during office hours, while family commitment and personal resting time were the main reasons for disagreeing with off-office hour appointments.

**Conclusion:**

We found that majority of our respondents chose to come early instead of arriving on time which disrupts the staggered appointment system and causes over crowdedness. Our findings also show that the majority of our respondents accept off-office hour appointments. This positive response suggests that off-office hour appointments may have a high uptake amongst the public and thus be a possible solution to distribute the patient load. Therefore, this information may help policy makers to initiate future plans to resolve congestions within public health care facilities which in turn eases physical distancing during the pandemic.

**Supplementary Information:**

The online version contains supplementary material available at 10.1186/s12913-021-07456-3.

## Background

Coronavirus Disease 2019 (COVID-19), a disease caused by Severe Acute Respiratory Syndrome Coronavirus 2 (SARS-CoV-2) is a global crisis affecting more than 200 countries, with more than 260 million confirmed cases and over five million deaths reported worldwide by the end of 2021 [1]. Physical distancing of one metre or more is an important measure to reduce person-to-person virus transmission [2]. However, this puts the Malaysian public healthcare system to a test as overcrowding is an issue even before the COVID-19 pandemic [3].

More than four years ago, staggered appointment was introduced to all public clinics to reduce the congestion. Under this staggered appointment system, patients are allocated specific appointment slots and they are required to arrive 30 min prior to the appointment time given [4]. Despite this introduction, implementing this system was an issue as it did not alleviate overcrowding in Malaysian public hospitals. In the midst of the pandemic, an additional method was looked into by the Ministry of Health to overcome facility congestions. A double shift system which consists of staffs working from 8 am to 5 pm and 12.30 pm to 9.30 pm in health clinics was introduced with the hopes of extending the operational hours of healthcare facilities [5, 6]. This double shift allows extension of healthcare services on weekdays to operate beyond normal office hours which in turn, opens up additional appointment slots to reduce healthcare facility congestion [7]. Apart from operating after office hours, weekend appointments may be an additional avenue that should be explored to further alleviate congestion.

Amidst this backdrop of implementations and suggestions, there exist a gap in policy recommendation in appointment systems for the public. There is no published evidence that cite the extent of acceptance that the public has towards such new arrangements by the government. Therefore, this study aims to gather the views of the general public towards a new clinic appointment system to accommodate physical distancing measures in the new normal. Having such information is crucial to gauge the demand of these services so that stakeholders can truly assess whether such a move is feasibly practical for the service end users.

## Methods

### Study setting and population

This was a cross-sectional anonymous web-based survey conducted between 10th September 2020 and 30th November 2020. Data were collected and managed using the Research Electronic Data Capture (REDCap) platform hosted at Clinical Research Centre, Hospital Pulau Pinang, Pulau Pinang, Malaysia [8, 9]. Snowball sampling method was used to recruit the respondents for this study by distributing the survey link to the research team’s personal and professional network as well as on social media platforms such as Facebook, Instagram, Twitter, LinkedIn, WhatsApp, and Telegram. The sample size was calculated with z-score of 1.96, margin of error of 0.05 and expected proportion of 50%, which gives us a sample size of 363. We chose this expected proportion as there was no previous studies done related to our research question. By taking into consideration a 30% drop-out rate, the minimum sample size of 520 was needed. This survey was opened to Malaysians aged 18 years and above. Prior to accessing the questionnaire, respondents were asked eligibility screening questions, and only those who were eligible proceeded to answer the questionnaire. All respondents were informed of their anonymity and online consent was taken at the beginning of the survey.

### Study instrument

The questionnaire used in this study was first developed in English and later translated to Bahasa Malaysia and Mandarin. A group of healthcare workers currently practicing at a healthcare facility were consulted during the questionnaire development stage to ensure content validity of the questionnaire. Following this, face validity was done with ten respondents from the study population to ensure comprehensibility. The questionnaire was subsequently modified according to the feedback received. This process was repeated until there were no more modifications needed.

The questionnaire consisted of 3 sections whereby the first section focused on the respondents’ sociodemographic information on age, sex, ethnicity, education level, employment status and monthly household income. Total monthly household income was classified into three main groups according to the income threshold of Malaysian 2019 statistics. The categories are B40, M40 and T20 where the income threshold is below RM4,850, RM4,850 to RM10,970, and above RM10,971 respectively [10].

The second section asked the respondents to select the healthcare sector (public or private) that they utilize most of the time. All respondents were also asked to choose between “yes” / “no” / “not sure” if they were given any clinic or hospital appointment in the past. Respondents who answered “yes” would be asked to indicate if they were given a choice to choose their preferred appointment time, and if they had ever been given any appointment on a weekend or after 5 pm on a weekday in the past. Both these weekend and after 5 pm weekday appointments are referred to as off-office hour appointments.

The final section started with a hypothetical scenario to explore respondents’ preferred arrival time interval should they be given a clinic/hospital appointment at a public healthcare facility on a weekday, between 10 and 11am. The options given started as early as 7.00am, with every half an hour time interval. The answers were later categorized into early (7.00am – 9.30am), on time (9.30am – 10.00am) or late (10.00am onwards). All respondents were required to indicate the reasons of their chosen arrival time interval from a list of choices provided. This is followed by two more scenarios assessing the respondents’ agreement with off-office hour clinical appointment which covers the weekday evening and weekend appointment. The respondents were asked to choose between “agree” or “disagree” with respect to the given scenario. They were prompted for the reasons of their chosen answer from a list of choices provided.

### Statistical analysis

The responses were analysed using descriptive and inferential statistics. All categorical variables were expressed in frequencies and percentages, whereas the continuous variables were expressed in mean with standard deviation (SD), or median with interquartile range (IQR) depending on the data distribution. After we conducted the analysis, we decided to perform an additional inferential statistic using Pearson chi-square to determine if any association existed between the most utilized healthcare sector categories and their agreement to off-office hour appointments. The statistical analysis was done using R version 4.0.2. A p-value of 0.05 was considered as statistically significant.

### Ethical consideration

This study was registered under the National Medical Research Register (NMRR) and approved by the Medical Research Ethics Committee (MREC) (NMRR-20–1654-55,650). The participation in this survey was voluntary. No personal identifiers were collected in the survey. Consent was obtained when the respondents click on the “I agree and consent to participate” button after reading the consent form.

## Results

### Sociodemographic

The study received a total of 1,144 complete responses. The mean age was 41.4 ± 12.4 years with majority being female respondents (67.4%) and slightly more than half were of Malay ethnicity (51.1%). A bigger proportion of respondents had tertiary education (74.4%) and were fully employed (69.1%) (Table 1).

**Table 1:** Sociodemographic of study respondents

Sociodemographic characteristics	Results (*N* = 1,144)
Age in years, mean (± SD)	41.4 (12.4)
Sex, n (%)	
Male	373 (32.6)
Female	771 (67.4)
Ethnicity, n (%)	
Malay	584 (51.1)
Chinese	382 (33.4)
Indian	116 (10.1)
Others	62 (5.4)
Education level, n (%)	
Secondary education & below	71 (6.2)
Form 6 / A-Level / Pre-University / Certificate / Diploma	222 (19.4)
Tertiary education (Bachelor Degree or higher)	851 (74.4)
Employment status, n (%)	
Full time employment	790 (69.1)
Part time employment	25 (2.2)
Self-employed or Freelance	97 (8.5)
Unemployed	63 (5.5)
Student	39 (3.4)
Retired	130 (11.4)
Total monthly household income, n (%)	
B40	428 (37.4)
M40	492 (43.0)
T20	224 (19.6)
Healthcare sector most utilized, n (%)	
Public	636 (55.6)
Private	508 (44.4)

### Experience and utilization of healthcare facilities

A total of 636/1144 (55.6%) respondents reported that they utilized public healthcare facilities most of the time before the COVID-19 pandemic, which consists of public clinics, public hospitals and university hospitals. The majority (882/1144, 77.1%) claimed to have had a clinic or hospital appointment in the past. More than half of them (551/882, 62.5%) were given a choice to choose their own appointment time. Only a small proportion of them experienced having an appointment scheduled on the weekend (134/882, 15.2%) or after 5 pm on a weekday (51/882, 5.8%). Most of these off-office hour appointments were experienced by respondents who utilized private healthcare facilities most of the time [see Additional file 1].

### Preferred arrival time for a specific appointment slot

**Fig. 1:**
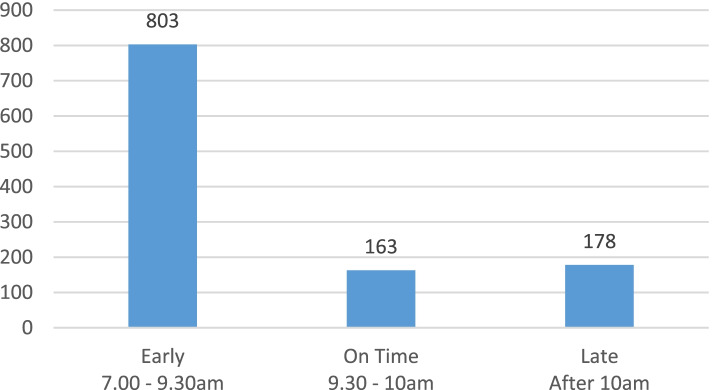
Respondents’ arrival time

In the first hypothetical scenario, respondents were asked to choose their preferred arrival time if they were given an appointment in a public healthcare facility on a weekday between 10-11am. 803 (70.2%) of them chose to arrive more than half an hour earlier. On the other hand, only 163 (14.2%) chose to arrive within half an hour (9.30-10am) before the appointment slot. This is depicted in Fig. 1. The top three reasons for the respondents to arrive early was due to parking difficulties, to shorten their time spent at the public healthcare facility and feeling that the registration process might take longer than expected (Table 2). Majority of those who selected to arrive within 30 min of the appointment chose the reason of shortening their time spent at the public clinic/hospital followed by difficult to get parking and registration time might take longer [ see Additional file 2]. However, 178 (15.6%) respondents chose to arrive after 10am for their appointment. The main reasons were due to work commitments, followed by the intention of shortening the time spent at the healthcare facility, and concern of getting a car park if arriving earlier [see Additional file 3].

**Table 2:** List of reasons chosen by the respondents for arriving early for the staggered appointment

Reasons of arriving at the government clinic / hospital more than half an hour earlier than the allocated appointment slot (*n* = 803)	Results, n (%)
Difficult to get parking	450 (56.0)
To shorten my time spent at the government clinic/hospital	385 (47.9)
The registration might take more time than I expected	336 (41.8)
I have a chance of seeing the doctor before 10am	281 (35.0)
Work commitments (e.g., So that I can get back to work on time)	269 (33.5)
I may not be able to see the doctor on time if I am late	256 (31.9)
I am worried that my appointment will be postponed/cancelled if I do not arrive on time	224 (27.9)
I depend on someone to drop me at the clinic/hospital	62 (7.7)
Difficult to get public transport	39 (4.9)
Other reasons	33 (4.1)
My physical condition may require extra time to be assisted into the clinic/hospital	31 (3.9)

### Preference for off-office hour appointments

In scenario two and three, majority of the respondents agreed to have an appointment scheduled after office hours on weekdays (723/1144, 63.2%) and weekends (798/1144, 68.9%) (Table 3). The top three reasons for agreeing with both these off-office hour appointments were similar. Around 70% of the respondents said it was due to work commitments. Around 50% of them said it will be easier to find parking and felt that their waiting time to see the doctor will be shorter [see Additional file 4 and 5]. However, around one-third of the respondents disagreed on the off-office hour appointments and the reasons were mostly related to family commitments and personal leisure time [see Additional file 6 and 7].

**Table 3:** Off-office hour appointments and most utilized healthcare sectors

	**After office hours**		**Weekend**	
	Agree	Disagree	Agree	Disagree
Total N (%)	723 (63.2)	421 (36.8)	798 (69.8)	346 (30.2)
Private n (%)	353 (69.5%)	155 (30.5%)	398 (78.3%)	110 (21.7%)
Public n (%)	370 (58.2%)	266 (41.8%)	400 (62.9%)	236 (37.1%)
P-value*	< 0.01		< 0.01	

In our additional analysis, there is a significant association between the acceptance for off-office hour appointments and the respondents’ most utilized healthcare facilities (Table 3). We found that majority of the respondents who mostly utilized private healthcare had agreed with having off-office hour appointments in the public sector if given a choice.

It was also noted that 628/1144 (54.9%) of the respondents agreed on both the off-office hour appointments and 251/1144 (21.9%) of the respondents disagreed on both after 5 pm weekdays and weekend appointments [see Additional file 8]. Almost 70% of those who disagreed on both the off-office hour appointments were female gender, more than 70% were fully employed and majority of them were from B40 and M40 household income group [see Additional file 9]. The main reasons for the disagreement are due to family commitments and personal resting time [see Additional file 10].

## Discussion

This study shows that majority (70.2%) of the respondents chose to arrive early if they were given an appointment at the public healthcare facility. It is consistent with a study by an American academic medical centre, which found that 62% of 6,194 heart failure patients arrived earlier than the scheduled time at the clinic. The study also demonstrated that the patients tend to arrive progressively earlier as the day goes on [11]. This could be one of the contributing factors for overcrowding and long waiting time in the Malaysian public healthcare system [3, 12, 13], despite staggered appointments being implemented since 2016 [14]. Our study shows that parking issue is the top reason for most respondents choosing to come much earlier than the scheduled appointment. According to a study done in a primary healthcare setting, tendency to come earlier than the given appointment time will cause more patients and carers to come to the healthcare facility at the same time [12]. Moreover, some respondents thought they would be able to shorten their time spent in the healthcare facility or see the doctor quicker if they arrive earlier than the given time slot. All these factors result in longer waiting time for other patients who were on time, which subsequently leads to congestion in the healthcare facility. Physical distancing becomes a challenge during a pandemic in this situation. Hence, introducing more appointment slots outside of the  usual office hours for patients may ease congestion and aid in physical distancing.

Majority of our study respondents agreed for an off-office hour appointment, with a higher agreement for a weekend appointment rather than after office hours on a weekday. A similar finding was reported in London, whereby 165/264 (62.5%) cardiac and respiratory patients wanted an off-office hour appointment, and the weekend appointment were the most preferred arrangement [15]. It was shown in our study that work commitment is the most common reason for agreeing with off-office hour appointments, regardless if it was on after office hours on a weekday or weekend. However, it was noted that majority of the respondents who agreed on a new appointment system other than usual office hour appointments are those who mostly utilized the private healthcare facilities. This observation maybe due to off-office hour appointments being commonly provided by the private healthcare facilities. Hence, this group of respondents may find it easier to adapt into this new system in the public healthcare if given a choice [16, 17]. Although there is a demand for off-office hour appointments, the actual utilization during its implementation needs to be monitored. According to Donne A.J. et al., the default rate was higher in the weekday evening sessions, especially among the follow-up patients [18]. Much effort and resources need to be invested to run an off-office hour clinic. Furthermore, Malaysian Medical Association (MMA) has voiced out that a more detailed study should be conducted before implementing this new system as there are human resource concerns [19]. Therefore, regular monitoring and evaluation is needed after its implementation to ensure it is cost effective.

Our study showed there are some respondents who totally disagree with off-office hour appointments (21.9%). Family commitments and personal resting time were the main reasons for them to disagree with the off-office hour appointments. As majority of the respondents are female gender, this may have resulted in family commitment being cited as the main reason for disagreement as it generally relates to women. Traditionally, women have been playing the major role as wives, mothers and primary caregivers to elderly parents. Women in Malaysia spend more hours than men on domestic works and caregiving activities [20, 21]. Similarly, in a survey conducted among parents on off-office hour outpatient clinics for the paediatric population, appointments on Sunday and weekday evening were the least preferred. A few reasons include the care for other children, bedtime for the children, disruption of meal times, and others [22]. Another main reason that study respondents disagreed on off-office hour appointments was it takes up their personal time. More than 70% from 251 respondents who disagreed were fully employed and off-office hour is the only time for them to rest and enjoy their leisure activities.

To the best of our knowledge, this is the first study looking into publics’ acceptance on staggered and off-office hour’s clinic appointments in Malaysia. Despite the ongoing COVID-19 pandemic, we were able to obtain a quick view of the public’s opinion on the appointment system via online survey which prevented face-to-face survey distribution. Nevertheless, a few limitations are worth noting in this study. To begin with, we did not collect any personal identifiers from the respondents. So, it is impossible to know if we have received multiple responses from the same respondent. Secondly, this study used a snowball sampling method where there is no assurance of the sample representativeness and may contribute to sampling bias. This was observed in the gender and education sociodemographic characteristics. Our sample did not match the Malaysian population demographics within these two domains. The general public demographics according to Department of Statistics Malaysia showed that in 2020 the ratio for male and female population was almost evenly distributed [23]. Quick Facts 2018 Malaysia Educational Statistics of the national population showed that less than 25% had more than secondary education [24]. We oversampled female gender in this study and highly educated respondents. Hence, findings of our study may be biased towards the female gender and highly educated groups.  Using an online survey also had added limitations  as respondents without internet facilities or not being digitally savvy  may not have access to this survey. Since this is an online survey, some respondents may have a different understanding and interpretation of the questions asked and there is no one to clarify for them. Besides, we were unable to derive if there was any association between patients’ health status and their preferences with the clinic appointment structure as we did not collect the information on their co-morbidities. Some of the respondents may not even utilise public services. In addition, we have only given the respondents a hypothetical scenario but in reality, the situation or response from the public may vary.

Regardless, this study offers an insight to the views of the Malaysian public on a new clinic appointment structure to aid physical distancing especially during the pandemic. Nevertheless, in-depth future research is needed involving policy makers and relevant stakeholders to have a successful implementation of a new policy.

## Conclusion

We found that majority of our respondents chose to come early for their appointments instead of arriving on time. This behaviour disrupts the staggered appointment system and causes over crowdedness. However, our findings also show that the majority of our respondents accept off-office hour appointments. This positive response suggests that off-office hour appointments may have a high uptake amongst the public and thus be a possible solution to distribute the patient load into these additional new slots. Therefore, this information may help policy makers to initiate future plans to resolve congestions within public health care facilities which in turn eases physical distancing during the pandemic.

## Supplementary Information

**Additional file 1.** Association between Healthcare sector mostly utilized and arrangement of office-hour appointments in the past.

**Additional file 2.** List of reasons chosen by the participants for arriving on-time for the staggered appointment.

**Additional file 3.** List of reasons chosen by the participants for arriving late for the staggered appointment.

**Additional file 4.** Reasons for agreeing on after 5pm weekday appointments.

**Additional file 5.** Reasons for agreeing on weekend appointments.

**Additional file 6.** Reasons for disagreeing on after 5pm weekday appointments.

**Additional file 7.** Reasons for disagreeing on weekend appointments.

**Additional file 8.** Acceptance of off-office hour appointments.

**Additional file 9.** Demographics of total disagreement with off-office hour appointments.

**Additional file 10.** Reasons of disagreement with off-office hour appointments.

**Additional file 11.**

## Data Availability

The datasets used and analysed for this study are available from the corresponding author on reasonable request.

## Abbreviations

B40: Bottom 40% of the Malaysian household income
COVID-19: Coronavirus Disease 2019
IQR: Interquartile Range
M40: Middle 40% of the Malaysian household income
MCO: Movement Control Order
MoH: Ministry of Health
MREC: Medical Research Ethics Committee
NMRR: National Medical Research Register
REDCap: Research Electronic Data Capture
SARS-CoV-2: Severe Acute Respiratory Syndrome Coronavirus 2
SD: Standard Deviation
T20: Top 20% of the Malaysian household income
