# Supplementary material for: Appointment structure in Malaysian healthcare system during the COVID-19 pandemic: The public perspective
Source: BMC Health Serv Res. 2022 Feb 3;22:141. doi: 10.1186/s12913-021-07456-3 (PMC8811595; doi:10.1186/s12913-021-07456-3)
Supplement: Supplementary file 1 — Additional file 1. Association between Healthcare sector mostly utilized and arrangement of office-hour appointments in the past. [file 12913_2021_7456_MOESM1_ESM.docx]

**Additional file 1: Association between healthcare sector mostly utilized and arrangement of off-office hour appointment in the past**

| Arrangement of off-office hour appointment in the past | | Healthcare sector, n (%) | | p value |
| --- | --- | --- | --- | --- |
|  |  | Public | Private |  |
| Weekend (n = 882) | |  |  |  |
|  | Yes | 26 (4.9) | 108 (30.5) | <0.001 |
|  | No | 502 (95.1) | 246 (69.5) |  |
| Weekday after 5pm (n = 882) | |  |  |  |
|  | Yes | 21 (4.0) | 30 (8.5) | 0.005 |
|  | No | 507 (96.0) | 324 (91.5) |  |
