# Supplementary material for: Appointment structure in Malaysian healthcare system during the COVID-19 pandemic: The public perspective
Source: BMC Health Serv Res. 2022 Feb 3;22:141. doi: 10.1186/s12913-021-07456-3 (PMC8811595; doi:10.1186/s12913-021-07456-3)
Supplement: Supplementary file 2 — Additional file 2. List of reasons chosen by the participants for arriving on-time for the staggered appointment. [file 12913_2021_7456_MOESM2_ESM.docx]

**Additional file 2: List of reasons chosen by the participants for arriving on-time for the staggered appointment.**

| **Reasons of arriving at the government clinic / hospital on time (within half an hour earlier than the allocated appointment slot) (n = 163)** | **Results,**  **n (%)** |
| --- | --- |
| To shorten my time spent at the government clinic/hospital. | 83 (50.9) |
| Difficult to get parking. | 72 (44.2) |
| The registration might take more time than I expected. | 71 (43.6) |
| I am worried that my appointment will be postponed/cancelled if I do not arrive on time. | 65 (39.9) |
| I may not be able to see the doctor on time if I am late. | 59 (36.2) |
| Work commitments (e.g., So that I can get back to work on time). | 49 (30.1) |
| I have a chance of seeing the doctor before 10am. | 23 (14.1) |
| Difficult to get public transport. | 12 (7.4) |
| I depend on someone to drop me at the clinic/hospital. | 10 (6.1) |
| Other reasons. | 7 (4.3) |
| My physical condition may require extra time to be assisted into the clinic/hospital | 5 (3.1) |
