# Supplementary material for: Appointment structure in Malaysian healthcare system during the COVID-19 pandemic: The public perspective
Source: BMC Health Serv Res. 2022 Feb 3;22:141. doi: 10.1186/s12913-021-07456-3 (PMC8811595; doi:10.1186/s12913-021-07456-3)
Supplement: Supplementary file 3 — Additional file 3. List of reasons chosen by the participants for arriving late for the staggered appointment. [file 12913_2021_7456_MOESM3_ESM.docx]

**Additional file 3: List of reasons chosen by the participants for arriving late for the staggered appointment.**

| **Reasons of arriving at the government clinic / hospital later than the allocated appointment slot (n = 178)** | **Results,**  **n (%)** |
| --- | --- |
| Work commitments (e.g., So that I can get back to work on time). | 86 (48.3%) |
| To shorten my time spent at the government clinic/hospital. | 64 (36.0%) |
| Difficult to get parking. | 52 (29.2%) |
| I am worried that my appointment will be postponed/cancelled if I do not arrive on time. | 32 (18.0%) |
| The registration might take more time than I expected. | 28 (15.7%) |
| I may not be able to see the doctor on time if I am late. | 27 (15.2%) |
| Other reasons. | 17 (9.6%) |
| I depend on someone to drop me at the clinic/hospital. | 14 (7.9%) |
| I have a chance of seeing the doctor before 10am. | 8 (4.5%) |
| Difficult to get public transport. | 7 (3.9%) |
| My physical condition may require extra time to be assisted into the clinic/hospital | 5 (2.8%) |
