# Supplementary material for: Appointment structure in Malaysian healthcare system during the COVID-19 pandemic: The public perspective
Source: BMC Health Serv Res. 2022 Feb 3;22:141. doi: 10.1186/s12913-021-07456-3 (PMC8811595; doi:10.1186/s12913-021-07456-3)
Supplement: Supplementary file 4 — Additional file 4. Reasons for agreeing on after 5pm weekday appointments. [file 12913_2021_7456_MOESM4_ESM.docx]

**Additional file 4: Reasons for agreeing on after 5pm weekday appointments.**
