# Supplementary material for: Appointment structure in Malaysian healthcare system during the COVID-19 pandemic: The public perspective
Source: BMC Health Serv Res. 2022 Feb 3;22:141. doi: 10.1186/s12913-021-07456-3 (PMC8811595; doi:10.1186/s12913-021-07456-3)
Supplement: Supplementary file 5 — Additional file 5. Reasons for agreeing on weekend appointments. [file 12913_2021_7456_MOESM5_ESM.docx]

**Additional file 5: Reasons for agreeing on weekend appointments.**
