# Supplementary material for: Appointment structure in Malaysian healthcare system during the COVID-19 pandemic: The public perspective
Source: BMC Health Serv Res. 2022 Feb 3;22:141. doi: 10.1186/s12913-021-07456-3 (PMC8811595; doi:10.1186/s12913-021-07456-3)
Supplement: Supplementary file 8 — Additional file 8. Acceptance of off-office hour appointments. [file 12913_2021_7456_MOESM8_ESM.docx]

**Additional file 8: Acceptance of off-office hour appointments**

| **Weekend Appointments** | **Weekday after 5pm** | |  |
| --- | --- | --- | --- |
|  | Agree | Disagree | Total |
| Agree | 628 | 170 | 798 |
| Disagree | 95 | 251 | 346 |
| Total | 723 | 421 | 1144 |
