# Supplementary material for: Appointment structure in Malaysian healthcare system during the COVID-19 pandemic: The public perspective
Source: BMC Health Serv Res. 2022 Feb 3;22:141. doi: 10.1186/s12913-021-07456-3 (PMC8811595; doi:10.1186/s12913-021-07456-3)
Supplement: Supplementary file 9 — Additional file 9. Demographics of total disagreement with off-office hour appointments. [file 12913_2021_7456_MOESM9_ESM.docx]

**Additional file 9: Demographics of total disagreement with off-office hour appointments**

| **Sociodemographic characteristics (N = 251)** | | **n (%)** |
| --- | --- | --- |
| Age in years, man (±SD) | | 41.1 (12.8) |
| Sex, n (%) | |  |
|  | Male | 78 (31.1) |
|  | Female | 173 (68.9) |
| Race, n (%) | |  |
|  | Malay | 144 (57.4) |
|  | Chinese | 59 (23.5) |
|  | Indian | 35 (14.3) |
|  | Others | 13 (4.8) |
| Education level, n (%) | |  |
|  | Secondary education & below | 14 (5.6) |
|  | Form 6 / A-Level / Pre-University / Certificate / Diploma | 55 (21.9) |
|  | Tertiary education | 182 (72.5) |
| Employment status, n (%) | |  |
|  | Full time employment | 183 (74.1) |
|  | Part time employment | 3 (1.2) |
|  | Self-employed or Freelance | 11 (4.4) |
|  | Unemployed | 5 (2.0) |
|  | Student | 10 (4.0) |
|  | Retired | 36 (14.3) |
| Total monthly household income, n (%) | |  |
|  | B40 | 102 (40.6) |
|  | M40 | 116 (46.2) |
|  | T20 | 33 (13.1) |
| Preference of Healthcare sector utilisation, n (%) | |  |
|  | Public | 175 (69.7) |
|  | Private | 76 (30.3) |
