# Supplementary material for: Appointment structure in Malaysian healthcare system during the COVID-19 pandemic: The public perspective
Source: BMC Health Serv Res. 2022 Feb 3;22:141. doi: 10.1186/s12913-021-07456-3 (PMC8811595; doi:10.1186/s12913-021-07456-3)
Supplement: Supplementary file 10 — Additional file 10. Reasons of disagreement with off-office hour appointments. [file 12913_2021_7456_MOESM10_ESM.docx]

**Additional file 10: Reasons of disagreement with off-office hour appointments**

| **After office hours - Disagree reasons (n=251)** | **n (%)** |
| --- | --- |
| Family commitments (e.g. children/parents/spouse). | 150 (59.8) |
| Takes up my resting/personal time. | 127 (50.6) |
| I feel that other supportive health services  (e.g. blood taking, radiological services e.g. X-ray, CT scans, Ultrasound) will not be open after office hours. | 108 (43.0) |
| To avoid traffic jam after office hours. | 106 (42.2) |
| I do not want to travel after office hours. | 98 (39.0) |
| I feel unsafe. | 36 (14.3) |
| Difficulty in getting public transport. | 21 (8.4) |
| Other reasons. | 8 (3.2) |
|  |  |
| **Weekend - Disagree reasons (n=251)** | **n (%)** |
| I do not want to spend my weekend in a clinic / hospital. | 174 (69.3) |
| Family commitments (e.g. children/parents/spouse) | 154 (61.4) |
| Takes up my resting/personal time. | 151 (60.2) |
| I feel that other supportive health services (e.g. blood taking, radiological services e.g. X-ray, CT scans, Ultrasound) will not be available after office hours. | 95 (37.8) |
| To avoid traffic jam during peak hours on weekends. | 56 (22.3) |
| Difficulty in getting public transport. | 16 (6.4) |
| Other reasons. | 7 (2.8) |
