# Supplementary material for: Appointment structure in Malaysian healthcare system during the COVID-19 pandemic: The public perspective
Source: BMC Health Serv Res. 2022 Feb 3;22:141. doi: 10.1186/s12913-021-07456-3 (PMC8811595; doi:10.1186/s12913-021-07456-3)
Supplement: Supplementary file 11 — Additional file 11. [file 12913_2021_7456_MOESM11_ESM.pdf]

An Analysis of Its Impact on the Provision of Routine Clinical Care and Views Toward Transitioning to a New Normal within the Malaysian Healthcare System

Please choose your preferred language.

Silih pilih bahasa pilihan anda.

[illegible]

◯ English
◯ Bahasa Melayu
◯ [illegible]

Thank you for your interest in participating in this survey.

The purpose of this study is to study the views of the general public towards implementing staggered, after office hours, and weekend clinic appointments when adapting to the new normal resulting from the COVID-19 pandemic in Malaysia.

The whole process may take around 10 - 15 minutes.

If you are keen to proceed, please answer the following questions to determine your eligibility to participate in this study.

Terima kasih atas kesudian anda untuk menyertai kajian ini.

Tujuan kajian ini adalah untuk mengkaji pandangan masyarakat umum dalam pelaksanaan temu janji klinik secara berperingkat, temu janji selepas waktu pejabat dan pada hujung minggu ketika menyesuaikan diri dengan keadaan baru yang disebabkan oleh wabak COVID-19 di Malaysia.

Kajian ini akan mengambil masa sekitar 10 minit.

Sekiranya anda berminat untuk meneruskan, sila jawab soalan-soalan berikut untuk menentukan kelayakan anda menyertai kajian ini.

[illegible].

ELIGIBILITY SCREENING

Nationality

Kewarganegaraan

[illegible]

◯ Malaysian / Warganegara / [illegible]
◯ Non-Malaysian / Bukan Warganegara / [illegible]

Date of Birth

Tarikh Lahir

[illegible]

____

Age

Umur

[illegible]

____

YOU ARE ELIGIBLE.
[Please CONTINUE.]
Thank you for your participation.

ANDA LAYAK UNTUK MENYERTAI SOAL-SELIDIK INI.
[Sila TERUSKAN.]
Terima kasih atas kesudian anda untuk mengambil bahagian dalam soal-selidik ini.

[illegible]
[[illegible] [illegible].]
[illegible]

SORRY, YOU ARE NOT ELIGIBLE.
[Please DO NOT CONTINUE.]
You may close this window to exit the survey and no data will be collected.

We would like to apologise that you are not the participant that we are hoping for.
The following reason(s) maybe why you are not eligible for this survey:

- You are LESS THEN 18 years old OR
- You are NOT a Malaysian

THANK YOU

MINTA MAAF, ANDA TIDAK MEMENUHI KRITERIA UNTUK MENYERTAI SOAL -SELIDIK INI.
[JANGAN TERUSKAN.]
Anda boleh tutup laman web ini.

Kami ingin meminta maaf kerana anda bukan peserta yang kami ingin. Sebab-sebab berikut. Berikut adalah sebab-sebab yang berkemungkinan anda tidak layak mengikuti tinjauan ini:

- Anda KURANG DARIPADA 18 tahun ATAU
- Anda BUKAN WARGANEGARA Malaysia

TERIMA KASIH

[illegible]
[[illegible] [illegible].]
[illegible].

PATIENT INFORMATION SHEET AND CONSENT

The study is conducted and coordinated by the Institute for Clinical Research (ICR), National Institute of Health (NIH).

This survey aims to study the views of the general public towards implementing staggered, after office hours and weekend clinic appointments when adapting to the new normal resulting from the COVID-19 pandemic in Malaysia.

This online survey will take about 10 minutes.

You may not copy, reproduce, distribute, publish, display, perform, modify, create derivative works, transmit, or in any way exploit any such content, nor may you distribute any part of this content over any network, sell or offer it for sale, or use such content to construct any kind of database. Copying or storing any content is prohibited without prior written permission of the Institute for Clinical Research.

All responses are anonymous and will be kept strictly confidential. We have attached the Patient Information Sheet in this section. If you would like to know more details about this study, you may download and go through the document below before proceeding to the consent.

[Attachment: "STANCE - PIS (English) Version 1.0.pdf"]

Kajian ini dijalankan dan diselaraskan oleh Institut Penyelididkan Klinikal (ICR), Institut Kesihatan Negara (NIH).

Tujuan kajian ini adalah untuk mengkaji pandangan masyarakat umum dalam pelaksanaan temu janji klinik secara berperingkat, temu janji selepas waktu pejabat dan pada hujung minggu ketika menyesuaikan diri dengan keadaan baru yang disebabkan oleh wabak COVID-19 di Malaysia.

Kajian ini akan mengambil masa sekitar 10 minit.

Anda tidak boleh menyalin, mengeluar semula, menyebar, menerbit, mempamer, melaku, mengubah suai, membuat karya terbitan, menghantar, atau dalam apa-apa cara mengeksploitasi mana-mana kandungan ini di mana-mana rangkaian, menjual atau menawar untuk dijual, atau meggunakan kandungan tersebut untuk mebina sebarang jenis pangkalan data. Menyalin atau menyimpan sebarang kandungan adalah dilarang tanpa kebenaran bertulis dari Institut Penyelidian Klinikal.

Kami telah melampirkan risalah maklumat pesakit di bahagian ini. Jika anda ingin mendapatkan maklumat yang selanjutnya tentang kajian ini, anda boleh muat turun dan baca dokumen ini sebelum memberikan persetujuan untuk menyertai kajian ini.

[Attachment: "STANCE - PIS (BM) Versi 1.0.pdf"]

(English)
• By selecting the "I AGREE AND CONSENT TO PARTICIPATE" button and submitting this form, you are indicating that:
- You have read and understood the information provided;
- You are over the age of 18;
- You agree to the terms as described.

○ I agree and consent to participate
○ I do not consent to participate

• Dengan menekan butang " SAYA SETUJU UNTUK MENYERTAI", ia menunjukkan bahawa anda:
- Telah membaca dan memahami semua informasi yang diberi;
- Berumur 18 tahun ke atas;
- Bersetuju dengan syarat seperti yang tertera di atas.

○ Saya setuju untuk menyertai.
○ Saya tidak setuju untuk menyertai.

•[illegible]"[illegible]"[illegible]
-[illegible]
-[illegible]18[illegible]
-[illegible]

○ [illegible]
○ [illegible]

Personal Information

Please answer the following questions about yourself.

GENDER

- ◯ Male
- ◯ Female

RACE

- ◯ Malay
- ◯ Chinese
- ◯ Indian
- ◯ Others

If "Others", please specify: ______________________

Where do you CURRENTLY LIVE?

- ◯ Johor
- ◯ Kedah
- ◯ Kelantan
- ◯ Melaka
- ◯ Negeri Sembilan
- ◯ Pahang
- ◯ Perak
- ◯ Perlis
- ◯ Pulau Pinang
- ◯ Sabah
- ◯ Sarawak
- ◯ Selangor
- ◯ Terengganu
- ◯ Wilayah Persekutuan Labuan
- ◯ Wilayah Persekutuan Putrajaya
- ◯ Wilayah Persekutuan Kuala Lumpur

HIGHEST LEVEL OF FORMAL EDUCATION

- ◯ No formal education
- ◯ Primary education (up to standard 6)
- ◯ Secondary education (up to form 5)
- ◯ Form 6 / certificate / diploma / A-level / Pre-university course
- ◯ Tertiary education (Degree, Master, PhD, DrPH)
- ◯ Others

If "Others", please specify: ______________________

EMPLOYMENT STATUS

- ◯ Paid employment, full time (8 hours of work a day)
- ◯ Paid employment, part time
- ◯ Self-employed or Freelance
- ◯ Student
- ◯ Unemployed
- ◯ Retired

What is your current JOB? ______________________

(E.g. Teacher, Manager, Engineer, Technician, Secretary, Doctor, IT consultant etc.)

TOTAL MONTHLY HOUSEHOLD INCOME

- ◯ Less than RM 2,500
- ◯ RM 2,501 to RM 4,850
- ◯ RM 4,851 to RM 10,970
- ◯ RM 10,971 and above

Patient Experience

Where do you seek medical treatment MOST OF THE TIME?

- ◯ Government clinic
- ◯ Government hospital
- ◯ Private clinic
- ◯ Private hospital
- ◯ University clinic / hospital (e.g. UMMC, HUKM, HUSM, HUITM)

Based on your answer above, have you ever UTILIZED the health facility since the beginning of MCO (Movement Control Order period (18th March onward)?

- ◯ Yes
- ◯ No

In your past experience, have you been given a CLINIC / HOSPITAL APPOINTMENT time?

(e.g. Appointment at 9am / Appointment at 10.30am etc)

- ◯ Yes
- ◯ No
- ◯ Not sure

Were you given a choice to CHOOSE your clinic / hospital appointment time?

- ◯ Yes
- ◯ No
- ◯ Not sure

Have you ever been given any clinic/hospital appointment AFTER 5 PM on a WEEKDAY?

- ◯ Yes
- ◯ No
- ◯ Not sure

Have you ever been given any clinic/hospital appointment on a WEEKEND?

(e.g. Saturday and Sunday, or Friday and Saturday for Johor, Kedah, Kelantan and Terengganu)

- ◯ Yes
- ◯ No
- ◯ Not sure

Appointment Slots

Imagine if you are given a CLINIC / HOSPITAL APPOINTMENT slot between 10am to 11am;

WHAT TIME would you MOST LIKELY arrive at the clinic / hospital?

Please choose one of the following options.

- ◯ 7.00 am - 7.30 am
- ◯ 7.30 am - 8.00 am
- ◯ 8.00 am - 8.30 am
- ◯ 8.30 am - 9.00 am
- ◯ 9.00 am - 9.30 am
- ◯ 9.30 am - 10.00 am
- ◯ 10.00 am - 10.30 am
- ◯ 10.30 am - 11.00 am
- ◯ After 11.00 am

WHY did you select this time?

Please select all that apply.

- ☐ So that my time spent at the clinic/hospital will be shorter.
- ☐ I have a chance of seeing the doctor before 10am.
- ☐ I may not be able to see the doctor on time if I am late.
- ☐ I am worried that my appointment will be postponed/cancelled if I do not arrive on time.
- ☐ My physical condition may require extra time to be assisted into the clinic/hospital
- ☐ The registration might take more time than I expected.
- ☐ Difficult to get parking.
- ☐ I depend on someone to drop me at the clinic / hospital.
- ☐ Difficult to get public transport.
- ☐ Work commitments (e.g. So that I can get back to work on time).
- ☐ Other reasons.

If "Other reasons", please specify:

____

After Work Hours

Imagine a clinic / hospital arranges your appointment AFTER 5 PM on a WEEKDAY;

Would you be happy with this arrangement?

- ◯ Yes
- ◯ No

WHY did you choose this option?

Please select all that apply.

- ☐ Ease of finding parking slots.
- ☐ Ease of getting transport.
- ☐ Lesser traveling time.
- ☐ I feel that I will have a longer consultation time with my doctor.
- ☐ I feel that my waiting time to see the doctor will be shorter.
- ☐ I feel that my waiting time to collect my medications from the pharmacy will be shorter.
- ☐ I feel that other supportive health services (e.g. blood taking, X-ray, CT scans, Ultrasound) will remain similarly available as during office hours.
- ☐ Does not take up my working hours.
- ☐ My family/carer can accompany me.
- ☐ Other reasons.

If "Other reasons", please specify:

____

WHY did you choose this option?

Please select all that apply.

- ☐ I do not want to travel after office hours.
- ☐ To avoid traffic jam after office hours.
- ☐ Difficulty in getting public transport.
- ☐ I feel unsafe.
- ☐ Takes up my resting/personal time.
- ☐ Family commitments (e.g. children/parents/spouse).
- ☐ I feel that other supportive health services (e.g. blood taking, radiological services e.g. X-ray, CT scans, Ultrasound) will not be open after office hours.
- ☐ Other reasons.

If "Other reasons", please specify:

____

Weekends

Imagine a clinic / hospital arranges your appointment on a WEEKEND;

(e.g. Saturday and Sunday or Friday and Saturday for Johor, Kedah, Kelantan and Terengganu)

Would you be happy with this arrangement?

- ◯ Yes
- ◯ No

WHY did you choose this option?

Please select all that apply.

- ☐ Ease of finding parking slots.
- ☐ Ease of getting transport.
- ☐ Lesser traveling time.
- ☐ I feel that I will have a longer consultation time with my doctor.
- ☐ I feel that my waiting time to see the doctor will be shorter.
- ☐ I feel that my waiting time to collect my medications from the pharmacy will be shorter.
- ☐ I feel that other supportive health services (e.g. blood taking, X-ray, CT scans, Ultrasound) will remain similarly available as during office hours.
- ☐ Does not take up my working hours.
- ☐ My family/carer can accompany me.
- ☐ Other reasons.

If "Other reasons", please specify:

____

WHY did you choose this option?

Please select all that apply.

- ☐ I do not want to spend my weekend in a hospital.
- ☐ To avoid traffic jam during peak hours on weekends.
- ☐ Difficulty in getting public transport.
- ☐ Takes up my resting/personal time.
- ☐ Family commitments (e.g. children/parents/spouse)
- ☐ I feel that other supportive health services (e.g. blood taking, radiological services e.g. X-ray, CT scans, Ultrasound) will not be available after office hours.
- ☐ Other reasons.

If "Other reasons", please specify:

____

Future Research

This is the end of the survey.

Thank you for your participation!

Would you be interested to participate in future research(s)? ◯ Yes ◯ No

If "Yes" , kindly leave your EMAIL address. ________________________

Maklumat Peribadi

Sila jawab soalan berikut mengenai diri anda.

JANTINA
- ◯ Lelaki
- ◯ Perempuan

BANGSA
- ◯ Melayu
- ◯ Cina
- ◯ India
- ◯ Lain-lain

Jika "Lain-lain", sila nyatakan: __________________________________

Di manakah anda TINGGAL SEKARANG?
- ◯ Johor
- ◯ Kedah
- ◯ Kelantan
- ◯ Melaka
- ◯ Negeri Sembilan
- ◯ Pahang
- ◯ Perak
- ◯ Perlis
- ◯ Pulau Pinang
- ◯ Sabah
- ◯ Sarawak
- ◯ Selangor
- ◯ Terengganu
- ◯ Wilayah Persekutuan Labuan
- ◯ Wilayah Persekutuan Putrajaya
- ◯ Wilayah Persekutuan Kuala Lumpur

TAHAP PENDIDIKAN TERTINGGI
- ◯ Tiada pendidikan normal
- ◯ Sekolah Rendah (sampai darjah 6)
- ◯ Sekolah Menengah (sampai tingkatan 5)
- ◯ Tingkatan Enam / Sijil / Diploma / A-level / Pra-Universiti
- ◯ Pengajian Tinggi (Sarjana Muda, Sarjana, PhD, DrPH)
- ◯ Lain-lain

Jika "Lain-lain", sila nyatakan: __________________________________

STATUS PEKERJAAN
- ◯ Pekerjaan bergaji, sepenuh masa (8 jam bekerja sehari)
- ◯ Pekerjaan bergaji, separuh masa
- ◯ Bekerja sendiri atau Freelance
- ◯ Pelajar
- ◯ Tidak bekerja
- ◯ Pesara

Apakah PEKERJAAN anda sekarang? __________________________________

(Cth. Guru, Pengurus, Jurutera, Juruteknik, Setiausaha, Doktor, Perunding IT dll)

JUMLAH PENDAPATAN BULANAN ISI RUMAH
- ◯ Kurang daripada RM 2,500
- ◯ RM 2,501 hingga RM 4,850
- ◯ RM 4,851 hingga RM 10,970
- ◯ RM 10,971 dan ke atas

Pengalaman Pesakit

Di manakah anda SELALU mendapatkan rawatan perubatan?

- ◯ Klinik Kesihatan Kerajaan
- ◯ Hospital Kerajaan
- ◯ Klinik Swasta
- ◯ Hospital Swasta
- ◯ Klinik / Hospital Universiti (e.g. UMMC, HUKM, HUSM, HUITM)

Berdasarkan jawapan anda di atas, pernahkah anda MENGGUNAKAN kemudahan fasiliti kesihatan tersebut sejak permulaan PKP (Perintah Kawalan Pergerakan (18 Mac dan seterusnya)?

- ◯ Ya
- ◯ Tidak

Dalam pengalaman anda yang lalu, pernahkah anda diberi TEMUJANJI KLINIK / HOSPITAL?

(cth. Temujanji pada pukul 9 pagi / Temujanji pada pukul 10.30 pagi dll)

- ◯ Ya
- ◯ Tidak
- ◯ Tidak pasti

Adakah anda diberi pilihan untuk MEMILIH waktu temu janji klinik / hospital anda?

- ◯ Ya
- ◯ Tidak
- ◯ Tidak pasti

Pernahkah anda diberi temu janji oleh klinik / hospital SELEPAS jam 5 petang pada HARI KERJA?

- ◯ Ya
- ◯ Tidak
- ◯ Tidak pasti

Pernahkah anda diberi temu janji oleh klinik / hospital pada HUJUNG MINGGU?

(cth. Sabtu dan Ahad, atau Jumaat dan Sabtu untuk Johor, Kedah, Kelantan dan Terengganu)

- ◯ Ya
- ◯ Tidak
- ◯ Tidak pasti

Slot Temu Janji

Sekiranya anda diberi TEMU JANJI KLINIK / HOSPITAL di antara 10 pagi dan 11 pagi;

Bilakah WAKTU yang anda BERKEMUNGKINAN untuk tiba di klinik / hospital?

Sila pilih salah satu daripada pilihan berikut

- ◯ 7.00 am - 7.30 am
- ◯ 7.30 am - 8.00 am
- ◯ 8.00 am - 8.30 am
- ◯ 8.30 am - 9.00 am
- ◯ 9.00 am - 9.30 am
- ◯ 9.30 am - 10.00 am
- ◯ 10.00 am - 10.30 am
- ◯ 10.30 am - 11.00 am
- ◯ Selepas pukul 11.00 pagi

MENGAPAKAH anda memilih waktu ini?

Sila pilih semua yang berkenaan.

- ☐ Supaya masa saya di klinik / hospital akan menjadi lebih pendek.
- ☐ Saya mempunyai peluang untuk berjumpa doktor sebelum jam 10 pagi.
- ☐ Saya mungkin tidak dapat berjumpa doktor pada masa yang tepat jika saya lewat.
- ☐ Saya bimbang temu janji saya akan ditangguhkan / dibatalkan sekiranya saya tidak sampai tepat pada waktunya.
- ☐ Keadaan fizikal saya mungkin memerlukan lebih masa untuk dibantu ke klinik / hospital.
- ☐ Pendaftaran mungkin akan mengambil masa yang lebih daripada yang saya jangkakan.
- ☐ Sukar mendapatkan tempat letak kereta.
- ☐ Saya bergantung pada orang lain untuk membawa saya ke klinik / hospital.
- ☐ Sukar mendapatkan pengangkutan awam.
- ☐ Komitmen kerja (cth. Supaya saya dapat kembali bekerja tepat pada waktunya).
- ☐ Sebab-sebab lain.

Jika "Sebab-sebab lain", sila nyatakan:

____

Selepas Waktu Kerja

Sekiranya klinik / hospital mengatur temu janji anda SELEPAS jam 5 petang pada HARI KERJA;

Adakah anda berpuas hati dengan aturan waktu ini?

- ◯ Ya
- ◯ Tidak

MENGAPAKAH anda memilih pilihan ini?

Sila pilih semua yang berkenaan.

- ☐ Mudah mencari tempat letak kereta.
- ☐ Mudah mendapatkan pengangkutan.
- ☐ Masa perjalanan yang kurang.
- ☐ Saya merasakan bahawa masa perundingan dengan doktor saya akan lebih lama.
- ☐ Saya merasakan bahawa masa menunggu untuk berjum doktor akan lebih pendek.
- ☐ Saya merasakan bahawa masa menunggu untuk mengambil ubat di farmasi akan lebih pendek.
- ☐ Saya merasakan bahawa perkhidmatan kesihatan seperti pengambilan darah, sinar-X, imbasan CT, Ultrasound akan beroperasi sama seperti waktu pejabat.
- ☐ Ia tidak menjejaskan waktu kerja saya.
- ☐ Keluarga/penjaga boleh mengiringi saya.
- ☐ Sebab-sebab lain

Jika "Sebab-sebab lain", sila nyatakan:

____

MENGAPAKAH anda memilih pilihan ini?

Sila pilih semua yang berkenaan.

- ☐ Saya tidak ingin melakukan perjalanan selepas waktu pejabat.
- ☐ Untuk mengelakkan kesesakan lalu-lintas selepas waktu pejabat.
- ☐ Sukar untuk mendapatkan pengangkutan awam
- ☐ Saya berasa tidak selamat.
- ☐ Ia mengambil masa rehat / peribadi saya.
- ☐ Komitmen keluarga (cth. anak/ibu-bapa/suami/isteri).
- ☐ Saya merasakan bahawa perkhidmatan kesihatan seperti pengambilan darah, sinar-X, imbasan CT, Ultrasound tidak akan beroperasi selepas waktu pejabat.
- ☐ Sebab-sebab lain

Jika "Sebab-sebab lain", sila nyatakan:

____

Hujung Minggu

Sekiranya klinik / hospital mengatur temu janji anda pada HUJUNG MINGGU;

(cth. Sabtu dan Ahad, atau Jumaat dan Sabtu untuk Johor, Kedah, Kelantan dan Terengganu)

Adakah anda berpuas hati dengan aturan waktu ini?

- ◯ Ya
- ◯ Tidak

MENGAPAKAH anda memilih pilihan ini?

Sila pilih semua yang berkenaan.

- ☐ Mudah mencari tempat letak kereta.
- ☐ Mudah mendapatkan pengangkutan.
- ☐ Masa perjalanan yang kurang.
- ☐ Saya merasakan bahawa masa perundingan dengan doktor saya akan lebih lama.
- ☐ Saya merasakan bahawa masa menunggu untuk berjum doktor akan lebih pendek.
- ☐ Saya merasakan bahawa masa menunggu untuk mengambil ubat di farmasi akan lebih pendek.
- ☐ Saya merasakan bahawa perkhidmatan kesihatan seperti pengambilan darah, sinar-X, imbasan CT, Ultrasound akan beroperasi sama seperti waktu pejabat.
- ☐ Ia tidak menjejaskan waktu pejabat saya.
- ☐ Keluarga/penjaga boleh mengiringi saya.
- ☐ Sebab-sebab lain.

Jika "Sebab-sebab lain", sila nyatakan:

____

MENGAPAKAH anda memilih pilihan ini?

Sila pilih semua yang berkenaan.

- ☐ Saya tidak ingin menghabiskan waktu hujung minggu saya di hospital.
- ☐ Untuk mengelakkan kesesakan lalu-lintas ketika waktu puncak pada hujung minggu.
- ☐ Sukar untuk mendapatkan pengangkutan awam.
- ☐ Ia mengambil masa rehat / peribadi saya.
- ☐ Komitmen keluarga (cth. anak/ibu-bapa/suami/isteri).
- ☐ Saya merasakan bahawa perkhidmatan kesihatan seperti pengambilan darah, sinar-X, imbasan CT, Ultrasound tidak akan beroperasi selepas waktu pejabat.
- ☐ Sebab-sebab lain.

Jika "Sebab-sebab lain", sila nyatakan:

____

Penyelidikan Masa Depan

This is the end of the survey.

Adakah anda berminat untuk mengambil bahagian dalam penyelidikan masa depan? ○ Ya ○ Tidak

Jika "Ya", sila tinggalkan alamat E-MEL anda. __________________________________
